# Cutaneous mucormycosis confirmed using a *Mucorales*-specific monoclonal antibody: a case study

**DOI:** 10.3389/fmed.2026.1823222

**Published:** 2026-05-08

**Authors:** Alyssa C. Hudson, Ben Rymer, Shwetha Pradeep, Jeremy N. Day, Dora E. Corzo-León, Elizabeth R. Ballou, Andrew M. Borman, Christopher R. Thornton

**Affiliations:** 1 Royal Devon University Hospitals NHS Foundation Trust, Exeter, United Kingdom; 2 Medical Research Council Centre for Medical Mycology, University of Exeter, Exeter, United Kingdom; 3 UKHSA Mycology Reference Laboratory, Bristol, United Kingdom; 4 ISCA Diagnostics Limited, Truro, United Kingdom; 5 Biosciences, University of Exeter, Exeter, United Kingdom

**Keywords:** biomarker, cutaneous mucormycosis, lateral-flow test, monoclonal antibody, *Mucorales*

## Abstract

**Background:**

Mucormycosis is a highly aggressive and destructive angio-invasive infection caused by fungi of the order *Mucorales*. Diagnosis currently relies on lengthy and insensitive culture of biopsy specimens, as well as time-consuming histopathological examination of tissue samples, which lacks specificity.

**Case presentation:**

This case study describes an unusual presentation of cutaneous mucormycosis in an immunocompetent 49-year-old man following a traumatic farming injury to the hand. Despite appearing clinically well and having wounds that looked macroscopically clean after repeated debridement, the patient had a persistent infection caused by the *Mucorales* fungus, *Lichtheimia ramosa*. Diagnosis was delayed due to low clinical suspicion and routine homogenisation of biopsy samples, which reduced the sensitivity of conventional fungal culture and microscopy. Early surgical debridement combined with antifungal therapy was essential; however, treatment was complicated by amphotericin B–associated nephrotoxicity, necessitating a switch to posaconazole. The patient ultimately achieved wound healing with preserved hand function.

**Conclusion:**

The case demonstrates key challenges in diagnosing cutaneous mucormycosis, particularly in immunocompetent patients with environmentally contaminated wounds. The study highlights the need for improved awareness, optimal tissue handling, and more specific diagnostics for mucormycosis. We demonstrate the successful clinical use of a *Mucorales*-specific monoclonal antibody (mAb TG11), both as an immunohistochemistry stain and within a rapid lateral-flow device (LFD), to detect signature molecules of *Mucorales* infection in homogenised tissue samples, thereby improving the accuracy and speed of detection.

## Introduction

Mucormycosis is a rapidly progressive and difficult-to-diagnose invasive fungal infection (IFI) caused by filamentous fungi of the order *Mucorales*. As a diverse group of environmental moulds, they are found worldwide in soil and on decaying organic matter. The World Health Organisation (WHO) has ranked *Mucorales* species as high-priority fungal pathogens, highlighting the need for improved diagnostics (1). Mucormycosis primarily occurs in immunocompromised individuals, typically with poorly controlled diabetes mellitus, haematological malignancy, solid organ transplant, neutropenia, or corticosteroid use (2–8). The disease manifests as life-threatening rhino-orbito-cerebral, pulmonary, cutaneous, gastrointestinal, or disseminated infections (9). The global incidence of mucormycosis is rising, likely due to the growing populations of susceptible patients (5, 10). Cutaneous mucormycosis is the third most common clinical presentation and typically results from trauma or burns. Unlike the other forms of disease, cutaneous *Mucorales* infections tend to occur in immunocompetent individuals, although cutaneous infections following hematogenous dissemination in neutropenic patients have been reported (2, 3, 5, 8, 10).

Treatment of mucormycosis involves a combination of aggressive surgical debridement and adjunctive antifungal therapy (10–12). Despite best-practice treatment, overall mortality remains high at approximately 50% (2, 5, 8). The mortality rate for cutaneous disease is approximately 30%, with extensive surgery, including limb amputation, frequently required to control the infection (10). Due to the rapid progression and destructive nature of mucormycosis, early diagnosis to allow prompt initiation of appropriate treatment is crucial for improving survival and limiting tissue loss and disfigurement (11, 13, 14).

Diagnosis of mucormycosis currently relies on a combination of histopathology, culture, and direct microscopy of clinical samples (11). These methods have significant limitations: Histopathology and direct microscopy employ non-specific stains and require a skilled operator to identify characteristic *Mucorales* hyphal morphology; culture is time-consuming and lacks sensitivity (15–17). The sensitivity of culture and direct microscopy for *Mucorales* fungi is further reduced when samples are processed by homogenisation, a routine procedure used in most microbiology laboratories to enhance bacterial detection (18, 19).

Thornton et al. (20) recently developed a pan-*Mucorales*-specific murine IgG2b monoclonal antibody (mAb) named TG11. The extracellular polysaccharides (EPSs) detected by mAb TG11 are produced by all *Mucorales* fungi during hyphal growth and are not expressed by ungerminated spores (21), thereby enabling the detection of invasive infections. The mAb has been incorporated into a lateral-flow device (TG11-LFD) for the rapid detection of *Mucorales* EPSs in human bronchoalveolar lavage fluid (BALf) and serum (20, 22), and its potential as a *Mucorales*-specific immunohistochemistry stain has been demonstrated in an *ex vivo* mouse lung infection model (21).

Here, we present a case study of cutaneous mucormycosis in an immunocompetent patient following a traumatic injury with farming machinery and discuss the complex diagnostic, therapeutic, and reconstructive challenges associated with this rapidly progressive angio-invasive disease. The case study highlights numerous difficulties in diagnosing mucormycosis and the importance of appropriate tissue processing to enhance the detection of *Mucorales* infections. We explore novel solutions to these diagnostic challenges, reporting for the first time the use of the TG11-LFD test for *Mucorales*-specific antigen detection in homogenates of human tissue, as well as the use of mAb TG11 as a *Mucorales*-specific stain in immunohistochemistry.

## Case description

A 49-year-old right-handed male individual presented to the emergency department of a large acute teaching hospital 1 h after sustaining an extensive deep tissue injury to the volar surface of his right hand from a straw cutter (Table 1). He was otherwise fit and well, with a history of asthma managed with inhaled steroids, nasal polyps, and gout treated with allopurinol. He works as an engineer and owns a smallholding with horses and sheep.

**Table 1:** Timeline of key clinical, microbiological, and surgical events during admission.

Day	Key findings	Microbiology/diagnosis	Management
D0	Deep contaminated volar hand injury; tendon and nerve injury; RMF fracture	—	Tetanus vaccination; IV co-amoxiclav; EUA, washout, and debridement
D3	Malodorous wounds, necrosis	Tissue sent for MC&S	Repeat debridement
D5	Ongoing infection	Environmental bacteria isolated	Escalation to IV meropenem + IV metronidazole + PO doxycycline
D7–9	Wounds clinically clean	Swabs sent	Continued antibiotics
D10	Mould isolated from the D7 swab	Putative *Mucorales* fungus; sent to the reference laboratory	IV liposomal amphotericin B started; VAC therapy initiated; reconstruction delayed
D11	Wounds healthy	Extended fungal culture negative	Continue L-AmB
D12	Mould isolated from the D9 swab	Putative *Mucorales* fungus; sent to the reference laboratory	Ongoing antifungal therapy
D14	EUA with tissue sampling	Mucormycosis confirmed; *Lichtheimia ramosa* identified	Continue L-AmB; multidisciplinary review
D15	—	—	Acute kidney injury secondary to L-AmB
D16	—	—	Switch to oral posaconazole
D18	Infection confirmed but wounds improving	Persistent fungal elements	L-AmB stopped; posaconazole continued
D19	Further debridement	Viable fungus still present	Aggressive surgical clearance
D20	Wounds clean	—	Discharged on posaconazole + VAC
D40	Ready for reconstruction	No fungal growth	Further excision + ALT flap reconstruction
D82	Healed wounds	—	Flap division; posaconazole stopped
6 months	Full healing	—	Ongoing functional reconstruction planned

D, Day of hospital admission; EUA, Examination under anaesthesia; MC&S, Microscopy, culture, and sensitivity; RMF, Right middle finger; RIF, Right index finger; VAC, Vacuum-assisted closure therapy; L-AmB, Liposomal amphotericin B; ALT flap, Anterolateral thigh free flap.

**Figure 1:**
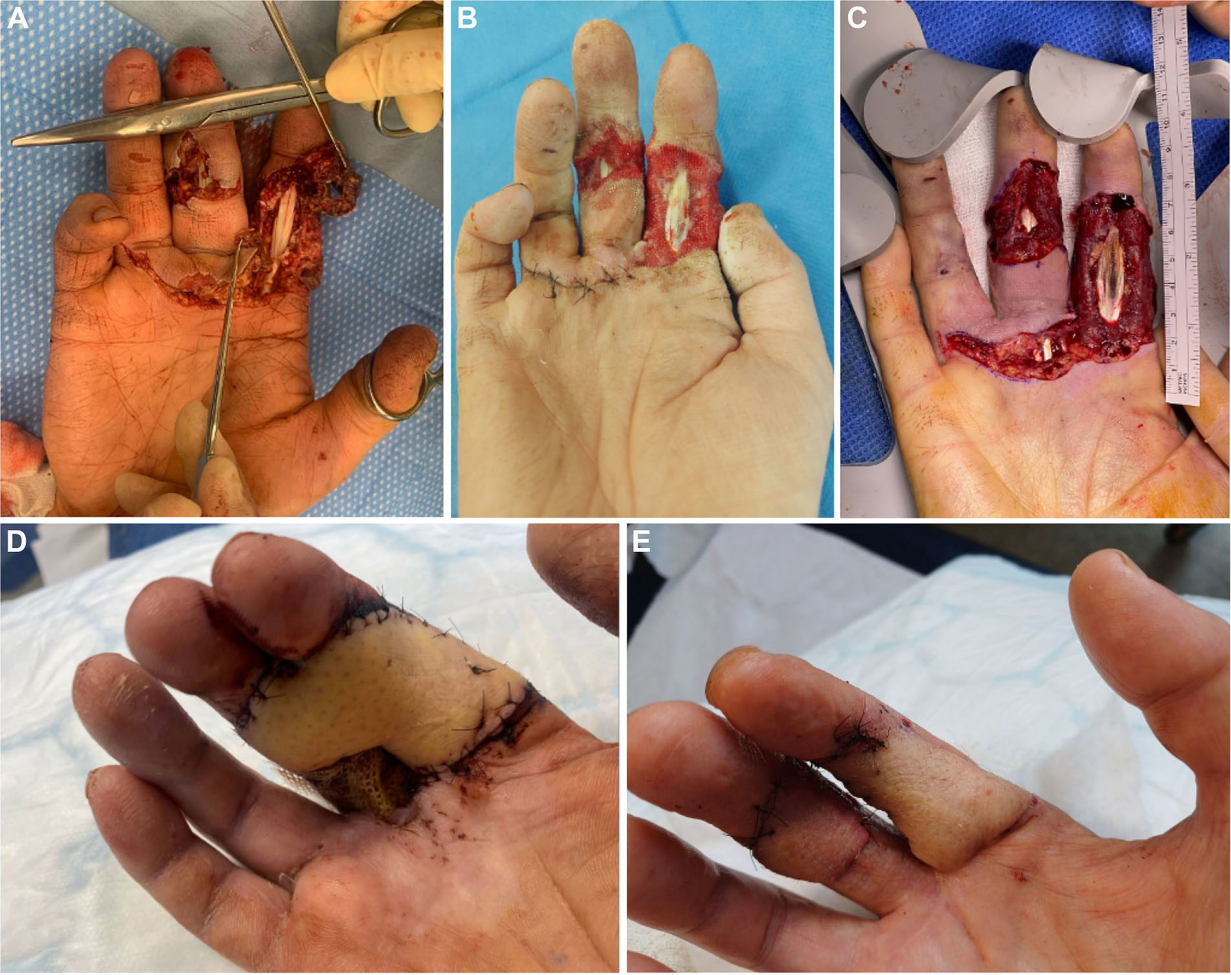
Right-hand wound presentation and surgical progression. **(A)** Complex volar hand wound at presentation. **(B)** Wound at D17 following five surgical debridements. Structures are exposed, but the wounds are clean despite ongoing microbiological and histopathological evidence of *Mucorales* infection. **(C)** Wound following oncological-style radical debridement (D19), with the aim of obtaining clean margins on histology and microscopy. **(D)** Wound closure with syndactylisation and a free ALT flap on D40. **(E)** Appearance following flap division and first-stage debulking on D82.

On admission, he was systemically well with stable observations. Blood tests showed mild neutrophilia and lymphopenia but were otherwise unremarkable. A tetanus vaccination was administered, and empiric intravenous (IV) amoxicillin-clavulanic acid was commenced. An X-ray revealed a displaced fracture of the proximal phalanx of the right middle finger (RMF). The wounds were assessed under examination under anaesthesia (EUA) and included a deep transverse laceration across the palm, as well as deep lacerations over the palmar aspect of the right index finger (RIF) and RMF with tendon exposure. There was a complete (100%) injury to the RMF flexor digitorum superficialis (FDS) and a 50% injury to the ulnar digital nerve (UDN) (Figure 1A). Primary surgical management involved wound debridement and thorough washout with normal saline.

The wounds were re-examined in theatre on day three (D3) and D5 of admission and were found to be malodorous, with tissue necrosis and turbid fluid. The wounds were further debrided and irrigated, and tissue was sent for microscopy, culture, and sensitivity (MC&S) at the local microbiology laboratory. Tissue samples were homogenised by bead beating prior to MC&S, in accordance with the standard local procedure. A wound swab was also sent on D5. Due to concerns regarding inadequately controlled infection, antibiotics were changed to IV meropenem, IV metronidazole, and oral doxycycline on D5. The wounds were re-examined on D7 and D9 and were found to be clean. Additional wound swabs were sent for MC&S. Tissue samples from D3 and D5 and a swab from D7 isolated a variety of environmental bacteria (*Bacillus cereus*, *Aeromonas eucrenophila*, *Enterococcus faecium*, *Clostridium sporogenes,* and other mixed anaerobes). Meropenem was discontinued after 4 days, and oral doxycycline and metronidazole were continued. Definitive reconstruction and wound closure with a flap were planned for D12.

**Figure 2:**
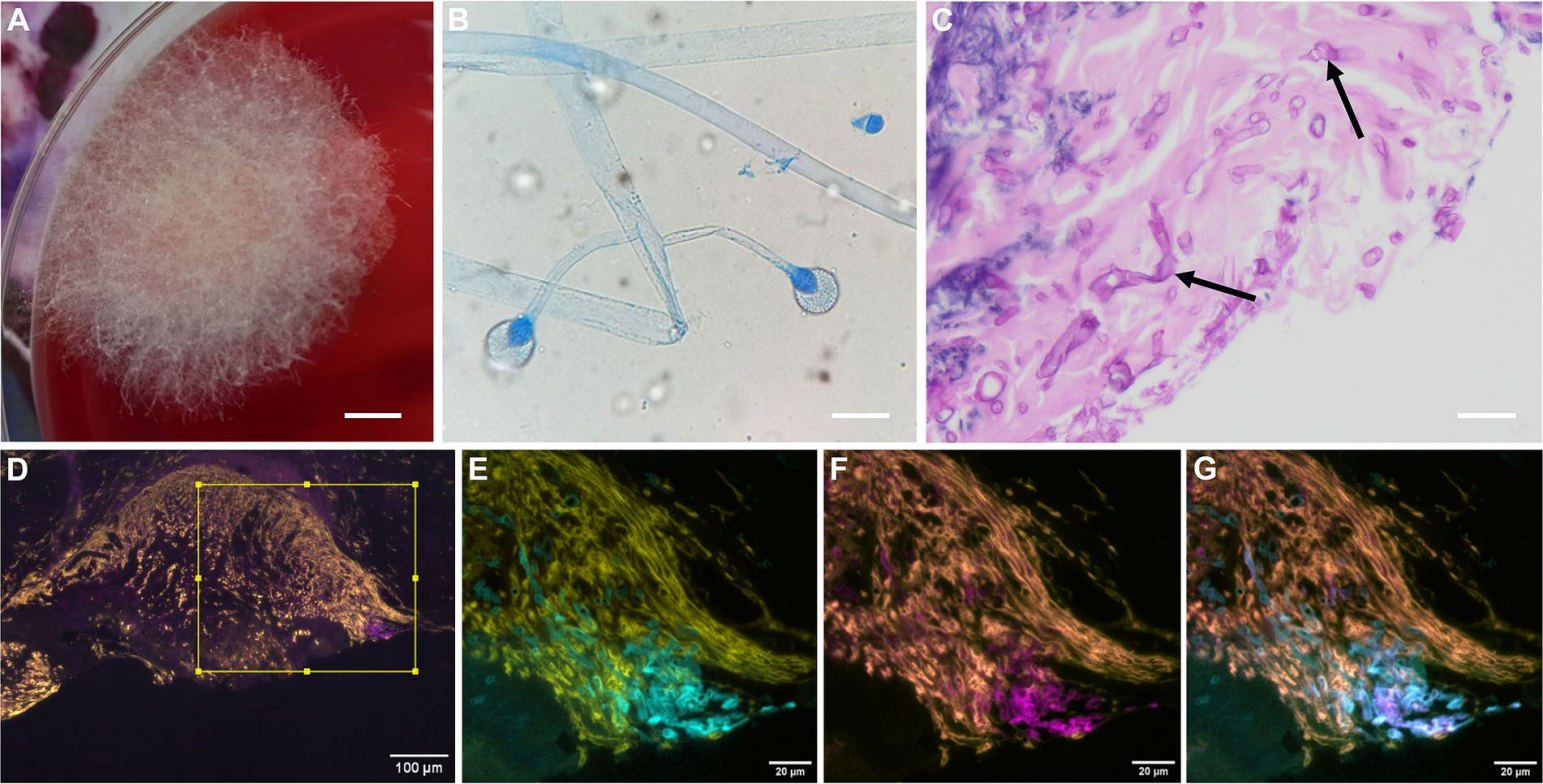
Microbiology, histopathology, and immunohistochemistry findings. **(A)** Fungal growth on blood agar after 48 h of incubation in CO_2_ at 37 °C, showing rapidly growing white floccose colonies with aerial hyphae. Scale bar = 0.4 cm. **(B)** Microscopy of the growing colony was performed using lactophenol cotton blue staining. Broad, pauci-septate, hyaline hyphae and sporangia were seen, typical of *Mucorales* fungi. Scale bar = 80 μm. **(C)** Formalin-fixed, paraffin-embedded tissue section with fungal elements stained with PAS-D (pink). Fragments of broad ribbon-like hyphae, indicative of *Mucorales* infection, were observed throughout (indicated by black arrows). Scale bar = 40 μm. **(D)** Section of D14 debrided tissue stained with propidium iodide, which stains DNA and host cell nuclei (yellow); calcofluor-white (CFW), which stains chitin in fungal cell walls (blue); and a monoclonal antibody (mAb) TG11 conjugated to the fluorophore Cyanine-5 (Cy5), which binds to extracellular polysaccharides (EPSs) specific to *Mucorales* fungi (magenta). **(E,F)** Higher-magnification images of the boxed region in **D**, showing CFW staining of fungal hyphae **(E)**, immunofluorescence of *Mucorales* hyphae with the TG11-Cy5 conjugate **(F)**, and an overlay of **E** and **F** demonstrating co-localisation of CFW staining with TG11-Cy5 immunofluorescence **(G)**. For immunohistochemistry, samples were deparaffinised and stained using the methodology described elsewhere (21).

However, on D10, a mould was isolated from the D7 swab sample and was presumptively identified as a putative *Mucorales* species based on colony growth and microscopic features (Figures 2A,B). The patient was immediately commenced on IV liposomal amphotericin B (L-AmB) at 5 mg/kg (D10), and plans for definitive surgery were delayed pending further investigation to establish the significance of the *Mucorales* mould. Vacuum-assisted closure (VAC) therapy was commenced to prevent desiccation of the exposed flexor tendons. The patient was investigated for underlying immunocompromise. He was HIV seronegative, and glycated haemoglobin (HbA1c) was within the normal range (39 mmol/mol). Repeat EUA on D11 showed the wound remained clean and healthy. Additional tissue samples were obtained for MC&S, including extended fungal culture (incubation at 37 °C on Sabouraud dextrose agar plus chloramphenicol (SABC) for 7 days). These samples were again homogenised prior to MC&S. Culture isolated only colonising bacterial flora; there was no fungal growth.

On D12, a putative *Mucorales* species was isolated from the D9 swab, and the organism was sent to the Mycology Reference Laboratory (MRL), Bristol, UK, for identification. Repeat EUA with tissue sampling was performed on D14, with samples sent directly to the MRL for direct microscopy and culture, as well as for local histological examination. Direct microscopy using calcofluor-white (CFW) staining revealed pauci-septate filamentous hyphae within the tissue parenchyma, indicative of mucormycosis. Similarly, broad, pauci-septate, ribbon-like hyphae were readily identified within the tissue parenchyma with Periodic Acid-Schiff with diastase (PAS-D) staining of histopathology samples (Figure 2C), confirming *Mucorales* infection. The *Mucorales* pathogen was isolated and identified as *Lichtheimia ramosa* using matrix-assisted laser desorption/ionisation time-of-flight mass spectrometry (MALDI-TOF MS; Mean LogScore = 2.11). Residual samples from D5 and D14 tissue debridements were sent to the Centre for Medical Mycology at the University of Exeter, where they were independently tested with a novel pan-*Mucorales*-specific mAb, TG11 (20), in immunohistochemistry (21) and by lateral-flow immunoassay (20).

Having confirmed the diagnosis of *Mucorales* infection on D18 of admission, the wound was re-examined and appeared clean, with no macroscopic evidence of necrosis and near-complete healing of the palmar wound (Figure 1B). A decision was made to postpone definitive reconstructive surgery until the infection had been adequately treated with surgical resection (aiming for clear margins) and adjunctive antifungal therapy. The three wounds were extensively debrided on D19 (Figure 1C), and two samples of clean tissue from each wound margin were sent to the MRL for MC&S and processed locally for histopathological examination. Sparse fragments of the pauci-septate filamentous fungus were visible on direct microscopy in four tissue samples. Fungal hyphae were identified on direct microscopy of tissue from the proximal RMF, and occasional fungal hyphae were evident on PAS-D staining of tissue from this site, although these were limited to superficial tissue layers. *Lichtheimia ramosa* was subsequently isolated on culture of tissue from the proximal RMF and right palm, demonstrating the ongoing presence of viable fungus in these wounds.

The patient’s treatment with adjunctive L-AmB was complicated by the development of acute kidney injury (AKI) on D15. Oral posaconazole 400 mg twice daily was commenced on D16, and L-AmB was subsequently discontinued on D18 following failure of renal function to improve. Therapeutic drug monitoring was performed throughout posaconazole therapy (D21, D28, and D59), and trough levels were consistently maintained above 1.0 mg/L. Anti-fungal susceptibility testing of the *L. ramosa* isolate using the CLSI broth microdilution methodology revealed minimum inhibitory concentrations (in mg/L) of 0.5, 0.5, and 0.25 for amphotericin B, isavuconazole, and posaconazole, respectively, suggesting susceptibility (or at least *in vitro* activity) to all three agents. His renal function spontaneously recovered following cessation of L-AmB, and he was discharged home on oral posaconazole with VAC therapy in place on D20 of admission.

The patient returned on D40 for definitive wound cover, as the wounds remained macroscopically clean, with the palmar wound already completely healed. The RIF flexor digitorum profundus was found to be non-viable and was excised. The RMF wound margin (where heavy fungal hyphae were last seen on microscopy) was further excised to ensure fungal clearance, and tissue was sent for histopathology and to the MRL for MC&S. The RIF and RMF wound edges were freshened and syndactylised along the contiguous wound margins, converting two wounds into one. A right suprafascial anterolateral thigh (ALT) flap was used to cover the now single wound (Figure 1D). This was anastomosed to the radial artery and cephalic vein in the anatomical snuffbox dorsally. Co-amoxiclav was administered for 48 h post-operatively, and oral posaconazole therapy was continued. No fungal elements were observed on direct microscopy, nor were any organisms isolated after 2 weeks of incubation. No evidence of fungal infection was seen on histopathological examination of tissue stained with PAS-D. The patient returned on D82 for division of the flap to the RIF and RMF and first-stage debulking (Figure 1E). Adjunctive posaconazole was discontinued.

At the 6-month follow-up, his hand had completely healed and the flap was well settled. He is awaiting further surgery to improve hand function.

## Discussion

This case study of cutaneous mucormycosis is unusual due to the mode of acquisition, the infecting species, and the clinical evolution of the disease. Cutaneous mucormycosis typically occurs as a result of penetrating trauma and burns following natural disasters such as tornadoes and tsunamis, as well as after sustaining combat-related blast injuries (23). The disease is rarely reported following farming accidents specifically (2, 10), despite these injuries having a high risk of contamination with environmental material and fungal spores. The *Mucorales* species most commonly implicated in cutaneous disease are *Apophysomyces* and *Saksenaea* (23). *Lichtheimia* species typically cause pulmonary mucormycosis and are rarely reported as agents of cutaneous infections (24). Infections typically progress rapidly, causing local tissue invasion with resulting infarction and necrosis (2, 10). In this case study, the wounds were initially aggressively debrided and appeared macroscopically clean and healthy from D7 onwards. However, viable fungus continued to be isolated from the wound, with evidence of ongoing tissue invasion on direct microscopy and histopathology up to 12 days later. Remarkably, the palmar wound had healed despite the continued presence of viable fungus at that site.

### Sample processing impacts fungal culture

This case study presented numerous diagnostic challenges. The UK Standards for Microbiological Investigation (19) recommends that, in suspected fungal infection, tissue samples should not be homogenised and that fungal culture on SABC and microscopy using the fungal brightener CFW should be performed (19). Tissue homogenisation is a standard procedure used to enhance bacterial microscopy and culture yield, but it limits culture recovery and microscopy detection of filamentous fungi by damaging fragile hyphae, especially those of *Mucorales* fungi (18). Dicing is the preferred method of tissue preparation for fungal detection (18). However, in our case, IFI was not suspected as a possible cause of the uncontrolled wound infection, primarily due to the patient being immunocompetent and non-diabetic. As a result, tissue samples were routinely processed in the local microbiology laboratory by homogenisation, and fungal culture and direct microscopy for fungal elements were not initially performed. Our case highlights how optimal processing of tissue samples for fungal detection in microbiology laboratories relies on clinical suspicion of fungal infection and awareness among laboratory staff to avoid routine homogenisation of tissue specimens. Furthermore, this case emphasises the importance of considering mucormycosis in individuals with environmentally contaminated wounds, even in the absence of traditional risk factors for IFI.

### Immunohistochemistry improves the accuracy of diagnosis

IFI was not considered in this case until a fungus was unexpectedly isolated from a wound swab taken in theatre and identified in culture as a putative *Mucorales* species. Even then, the significance of this isolate was uncertain as it may simply reflect wound colonisation with *Mucorales* spores or culture contamination. Additional tissue samples were collected on D11 and cultured specifically for fungal pathogens; however, the tissues continued to be homogenised and, likely because of this, fungal culture was negative. All subsequent tissue samples were sent directly to the MRL for MC&S, where they were prepared by dicing for direct microscopy and culture. Direct microscopy with CFW was performed on the day of receipt, providing rapid confirmation of the presence of fungal hyphae within the tissue.

Visualisation of fungal elements in a normally sterile tissue biopsy, together with isolation and identification of the infecting species by microscopy, is considered the gold standard for diagnosing IFI. Histopathology and microscopy offer the advantage of enabling rapid detection of fungal pathogens, potentially within hours of sample receipt (25). However, stains used to visualise fungi in biopsy samples, such as CFW and PAS-D, are non-specific and cannot discriminate between *Mucorales* fungi and other environmental moulds that are also capable of causing cutaneous mycoses in immunocompetent individuals, such as *Aspergillus* (26, 27) and *Fusarium* (28) species.

To differentiate *Mucorales* fungi from other fungal pathogens, species-specific (29), genus-specific (23), or order-specific (20) reagents are needed. In our case study, we used a newly developed and commercially available pan-*Mucorales*-specific monoclonal antibody, mAb TG11, in immunohistochemistry (21) to confirm the presence of *Mucorales* hyphae in the D14 tissue debridement (Figures 2D–G), providing diagnostic specificity for cutaneous mucormycosis beyond that obtainable with non-specific fungal stains such as CFW and PAS-D.

### Rapid antigen testing improves the speed of diagnosis

This case of cutaneous mucormycosis was complex to treat not only because of acute kidney injury caused by amphotericin B but also because of the paucity of diagnostic tests that allow early and rapid diagnosis of mucormycosis and timely initiation of *Mucorales*-active antifungal therapy (amphotericin B, isavuconazole and posaconazole). The decision regarding when to perform definitive reconstruction was challenging, as it was time-critical due to the risk of tendon desiccation, but would likely have failed if infection remained uncontrolled, leading to progressive circumferential tissue necrosis.

While polymerase chain reaction (PCR) tests have been developed for the detection of circulating *Mucorales* nucleic acids in blood and BALf samples (22, 30), they are not widely available locally and instead rely on centralised, specialised laboratories. Furthermore, unlike direct microscopy, immunohistochemical staining, and culture, which are strongly recommended for mucormycosis detection, the use of PCR is only moderately recommended (11). Novel rapid diagnostics are urgently needed to facilitate prompt and accurate diagnosis of mucormycosis. The speed and simplicity of lateral-flow technology make it ideally suited for point-of-care testing of infectious diseases, meeting the ASSURED (affordable, sensitive, specific, user-friendly, rapid, equipment-free, and delivered) criteria for diagnostics, particularly in settings where access to sophisticated diagnostic procedures is limited (31).

**Figure 3:**
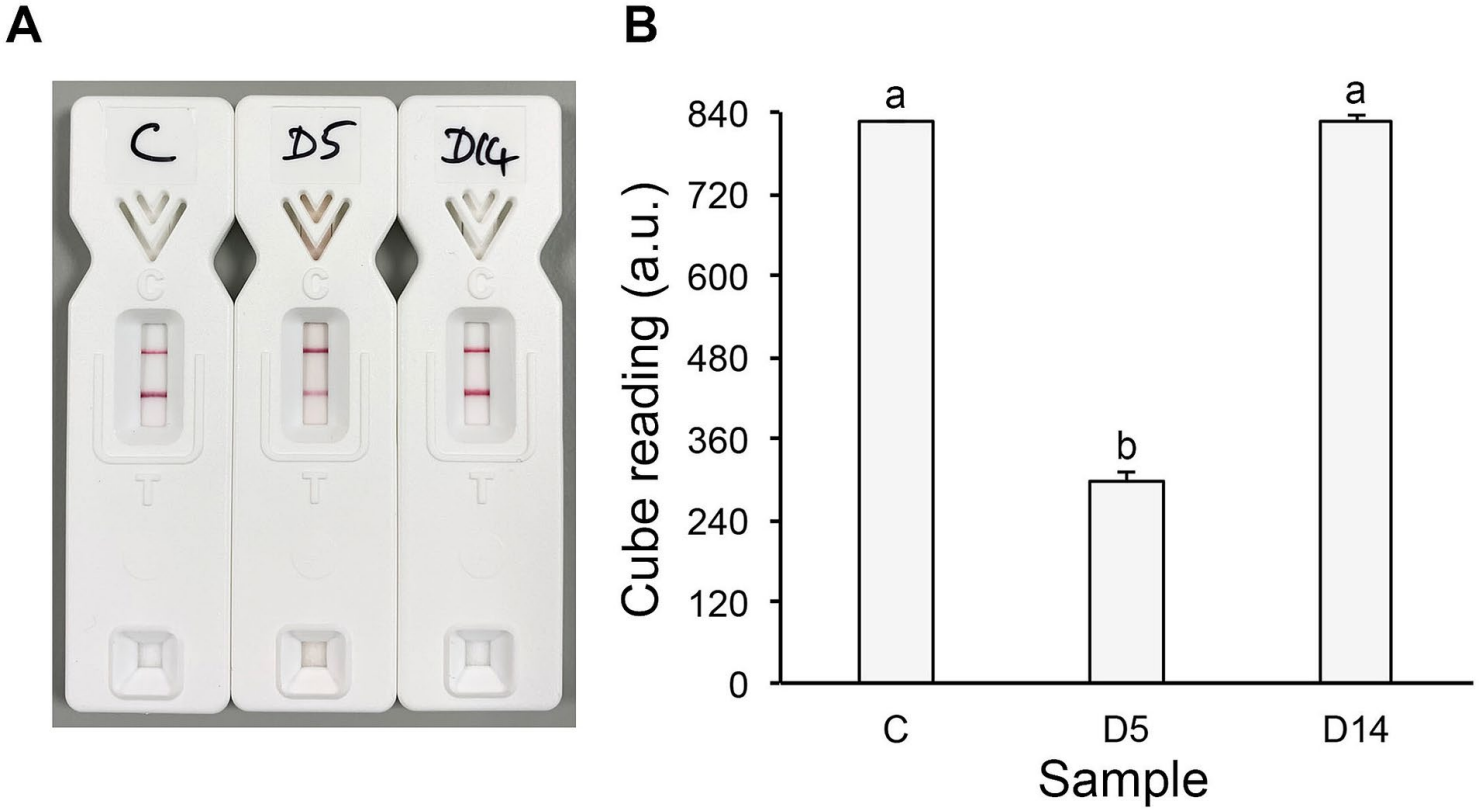
TG11-LFD tests of homogenised tissue samples from the D5 and D14 debridements. Samples were centrifuged at 17,000 *x g* for 5 min, and the aspirates were mixed 1:5 (vol:vol) with LFD running buffer (20). The experimental control (C) comprised a 1:5 (vol:vol) mixture of sterile dH_2_O and running buffer. One hundred μL of each mixture was applied to the TG11-LFD, and after 30 min, the intensities of the test (T) and internal control (C) lines were measured using a Cube reader in artificial units (a.u.) (20). The TG11-LFD is a competitive lateral-flow immunoassay in which the T line intensity is negatively correlated with the soluble analyte (*Mucorales* extracellular polysaccharide) in the homogenised tissue sample. Consequently, higher analyte levels produce weaker signals, whereas the absence of analyte results in the strongest signal (20). **(A)** Representative images of the TG11-LFD tests. There was an observable reduction in the test (T) line intensity in the D5 tissue homogenate sample compared to the experimental control (C), indicating the presence of a *Mucorales*-specific antigen in this sample. No reduction in the T line intensity was apparent in the D14 tissue homogenate sample compared to the experimental control, indicating the absence of the antigen in this latter sample. **(B)** There was a significant reduction in the LFD test (T) line intensity in the D5 tissue homogenate sample (mean a.u. value of 297.3 ± 11.8) compared to the experimental control (mean a.u. value of 826.5 ± 0.5), indicating the presence of the *Mucorales*-specific TG11 antigen in this sample. No significant difference in the mean T line intensity was seen in the D14 sample (a.u. value of 826.8 ± 6.8) compared to the experimental control, indicating the absence of the *Mucorales*-specific antigen in this latter sample. Bars represent the means of two technical replicates ± SE. Analysis of variance was used to compare means, and *post hoc* Tukey–Kramer analysis was used to determine statistical significance. Bars with different letters are significantly different from one another at *p* < 0.05. All LFD tests had mean internal control (C) line values of >600 a.u.

In this case study, we demonstrate the clinical utility of a newly developed rapid (30 min) antigen test, the TG11-LFD (20), for the detection of *Mucorales*-specific EPSs in an early (D5) homogenised tissue debridement (Figures 3A,B), thereby facilitating the prompt diagnosis of cutaneous mucormycosis based on a soluble diagnostic biomarker of active infection. Unlike *in situ* visualisation of *Mucorale*s hyphae in the D14 debrided tissue using mAb TG11 immunohistochemistry, the soluble TG11 diagnostic antigen was no longer detectable in the D14 homogenised sample, likely reflecting a reduction in the bioburden of the infection (and detectable secreted antigen levels) at this stage following successive debridement and initiation of antifungal therapy. Consequently, the TG11-LFD may have a valuable role in cases such as ours by allowing early diagnosis of cutaneous mucormycosis prior to anti-fungal treatment and in situations where tissue samples have already been processed by homogenisation.

## Conclusion

In conclusion, this case of cutaneous mucormycosis caused by *Lichtheimia ramosa* in an immunocompetent patient underscores the insidious nature of *Mucorales* infections, even when wounds appear clinically well and are repeatedly debrided. It highlights the critical importance of maintaining a high index of suspicion in environmentally contaminated traumatic injuries, optimising tissue handling to preserve fragile fungal elements, and ensuring early specialist microbiological input. The successful use of the pan-*Mucorales*-specific monoclonal antibody TG11, both in immunohistochemistry and within a rapid lateral-flow device, demonstrates the potential of targeted antigen detection to overcome limitations of conventional diagnostics, particularly when samples have been homogenised. Prompt recognition, aggressive surgical management, and appropriate antifungal therapy—balanced against drug-related toxicity—were pivotal in achieving infection control and functional limb salvage. Broader implementation of rapid, specific diagnostic tools may facilitate earlier diagnosis, guide timely therapeutic decisions, and ultimately improve outcomes in this destructive and life-threatening infection.

## Patient perspective

From a patient’s perspective, my main concern was the wounds and their repair and recovery. It is only on reflection that you realise that those who deliver news about problems in the wound are, in fact, saving your life. The incredible work from Alyssa and her team, and the reconstruction from Ben and his team, means that I have a life and can reflect on the moment of misjudgement when the accident happened and feel proud to have supported developments that might help save someone else in the future.

## Data Availability

The raw data supporting the conclusions of this article will be made available by the authors, without undue reservation.
